# ^13^C NMR Reveals No Evidence of n−π* Interactions in Proteins

**DOI:** 10.1371/journal.pone.0042075

**Published:** 2012-08-02

**Authors:** Bradley Worley, Georgia Richard, Gerard S. Harbison, Robert Powers

**Affiliations:** Department of Chemistry, University of Nebraska-Lincoln, Lincoln, Nebraska, United States of America; Instituto de Investigación Sanitaria INCLIVA, Spain

## Abstract

An 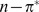 interaction between neighboring carbonyl groups has been postulated to stabilize protein structures. Such an interaction would affect the 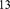
C chemical shielding of the carbonyl groups, whose paramagnetic component is dominated by 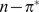 and 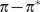 excitations. Model compound calculations indicate that both the interaction energetics and the chemical shielding of the carbonyl group are instead dominated by a classical dipole-dipole interaction. A set of high-resolution protein structures with associated carbonyl 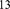
C chemical shift assignments verifies this correlation and provides no evidence for an inter-carbonyl 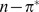 interaction.

## Introduction

Proteins exhibit a diversity of structures, with 1,282 folds or topologies present in the CATH database [1]. Each unique structure is defined by its amino acid composition, where sequence identities greater than 40% imply homologous structures [2]. Predicting the three-dimensional conformation of a protein from its primary sequence is a fundamental challenge of structural biology, and achieving this goal requires a thorough understanding of the underlying interactions and forces that stabilize protein structures [3].

Hydrophobic interactions and hydrogen bonds are two of the most common forces attributed to the overall stability of protein structures [4], [5]. The burial of hydrophobic residues is generally considered a major driving force in protein folding [6] and has been predicted to contribute roughly 8 kJ/mol per buried residue. Conversely, the contribution of hydrogen bonds to protein structure stability has been controversial [7]. Hydrogen bonds have been described as destabilizing, partially stabilizing or important driving forces. Of course, hydrogen bonds are a defining feature of 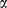-helices, 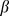-sheets and turns. Thus, the generally accepted view is that hydrogen bonds within a protein structure are marginally favored over hydrogen bonds to water. Hydrogen bonds are estimated to contribute roughly 4 kJ/mol to protein stability, but can vary based on the polarity of the microenvironment [8]. Despite these observations, a satisfying general mechanism for protein folding has not been described [9], [10]. This implies that our understanding of the factors involved in protein folding and stability is incomplete.

**Figure 1:**
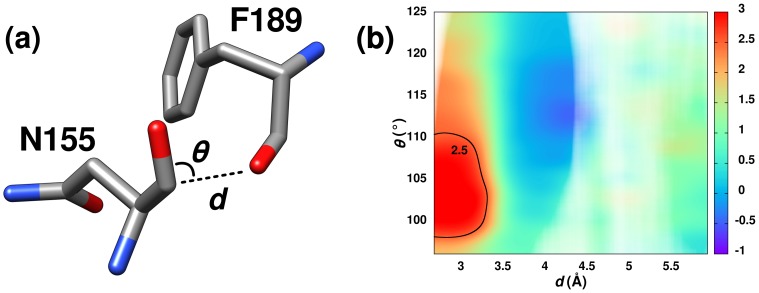
Predicted 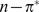 Interaction and Associated Carbonyl 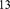
C Chemical Shifts. (**A**) Residues Asn155 and Phe189 from the x-ray structure of *Bacillus amyloliquefaciens* subtillisin BPN’ (PDB ID: 1v5i) illustrating the structural features for an optimal 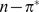 interaction between carbonyl groups. (**B**) 2D contour plot of carbonyl 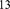
C chemical shift differences relative to random coil values as a function of the distance (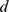) and angle (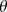) between carbonyls. A Gaussian smoothing function was applied to the data with 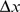 and 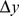 of 0.3 Å and 1.5°, respectively. A transparency mask based on the density of experimental data (see also Figure S1) is overlaid on the contour plot. Regions lacking experimental data are white. Positive values indicate downfield shifts.

In a recent paper, Bartlett *et. al.* proposed a new and potentially important interaction analogous to the hydrogen bond [11]. Unfortunately, the predicted 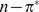 interaction was based on density functional theory and a relatively low-level basis set. However, conventional Kohn-Sham density functional theory does not properly model virtual orbitals [12] such as the 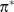 orbital proposed by Bartlett *et. al.* to have a role in protein stabilization. Moreover, the relatively low-level basis set used by the authors is inadequate to model such orbitals, and likely gives rise to substantial basis-set superposition errors. Also, experimental data in support of this prediction was not presented. Nevertheless, the predicted 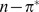 interaction was suggested to aid in the stabilization of protein structures and contribute roughly 0.4 to 5.4 kJ/mol. This stabilization is predicted to occur through the electron delocalization of the lone pair (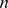) of a carbonyl oxygen atom to the antibonding orbital (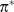) of a neighboring carbonyl carbon atom. An optimal 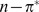 interaction was predicted to be restricted to a specific range of structural parameters (Figure 1A) corresponding roughly to the Bürgi-Dunitz trajectory [13]. The distance (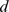) between the donor oxygen and acceptor carbon must be 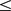
3.2 Å, and the angle between the (donor O)
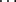
(acceptor C) vector and the acceptor carbonyl vector (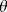) must lie between 99° and 119°. Interestingly, the structural parameters required for an optimal 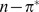 interaction are prevalent in a wide variety of common secondary structures, including 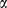-helices, 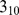-helices and twisted 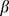-sheets, suggesting a potential alternative explanation.

Despite the presence of numerous conformations consistent with the 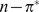 interaction in protein structures, no experimental evidence has been presented that supports the actual existence of this interaction. NMR chemical shifts of 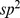-hybridized groups contain a paramagnetic component caused by mixing of excited states with non-zero orbital angular momentum into the diamagnetic ground state [14]. These excitations are predominantly 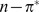 and 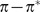 and are therefore highly sensitive to perturbations of these orbitals. The predicted 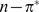 interactions between neighboring carbonyls would be expected to modify the local electronic environment of the acceptor carbonyl carbon nucleus, and the NMR 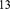
C chemical shift of the acceptor carbonyl carbon would experience a significant chemical shift change in the presence of an 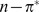 interaction [15]. A large (roughly 20 ppm range) linear relationship has been previously observed between carbonyl 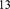
C chemical shifts and the carbonyl 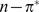 transition energy [16], [17].

An extensive analysis of 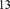
C chemical shifts correlated to high-resolution x-ray structures combined with a detailed analysis of the molecular orbitals of a formamide trimer model complex does not support the postulated 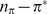 interaction. In fact, our model indicates that an 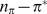 interaction is implausible. Instead, a simple dipole-dipole interaction better explains the observed effects used in support of the 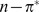 interaction. A prior manuscript by the same authors dismissed the dipole-dipole interaction explanation without elaboration [18].

## Results

A total of 2,516,360 residue pairs from a set of 164 high-resolution (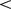
1.6 Å) x-ray crystal structures with assigned and uniformly referenced carbonyl 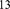
C chemical shifts were analyzed for potential 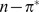 interactions. Setting a maximal distance of 6.0 Å between the donor oxygen and acceptor carbon yielded 45,792 potential acceptor carbonyl carbon atoms. The carbonyl 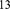
C chemical shift differences relative to random coil values for each of the 45,792 potential acceptor carbonyls were plotted against the 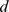 and 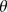 values for each carbonyl pair (Figure 1B). These chemical shift differences represent the contribution from the local structural environment, and potentially the 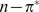 interaction. The two-dimensional contour plot indicates a maximal downfield shift of 2.9 ppm centered on the optimal structural parameters predicted for an 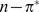 interaction.

Of the 45,792 carbonyls, 5,378 had optimal 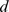 and 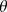 values for an 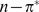 interaction and 40,414 were outside this optimal range. The mean carbonyl 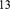
C chemical shift difference for the 40,414 carbonyls labeled as non-interactors is 0.58 ± 1.98 ppm. In contrast, the mean carbonyl 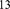
C chemical shift difference for the 5,378 interactors is 2.93 ± 2.41 ppm. A Student’s t-test indicates the difference of 2.35 ppm between the two means is statistically significant at the 99.9% confidence level. To address possible errors introduced into the analysis by highly dynamic residues in the x-ray structures, possible carbonyl interactors with B-factors greater than two standard deviations above the mean were omitted from the dataset. In the resultant set of 44,302 potential carbonyl interactors, the 2.33 ppm chemical shift difference was statistically indistinguishable from the original analysis. Similarly, possible interactors predicted at a 95% confidence level to participate in crystal-packing interfaces were also omitted from the dataset. Again, the corresponding set of interactors yielded a chemical shift difference of 2.33 ppm, which is still statistically significant at the 99.9% confidence level.

To address the possiblity that differences between x-ray crystal structures and NMR solution structures may lead to errors in the analysis, a replicate analysis was performed on a set of 137 NMR solution structures corresponding to the same set of 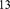
C and 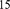
N chemical shifts used previously. Structural alignments using MAMMOTH showed a mean rmsd of 1.87 ± 0.57 Å between the pairs of x-ray and NMR structures. Of the 1,419,547 resulting carbonyl pairs from the NMR structures, 38,534 pairs were found to be potential interactors. Of the carbonyls in that set, 2,510 interactors were found with a mean carbonyl 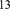
C chemical shift difference of 2.84 ± 1.71 ppm. The remaining 36,024 non-interactors had a mean carbonyl 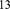
C chemical shift difference of 1.02±2.02 ppm. Again, the 1.82 ppm difference between the two means is statistically significant at the 99.9% confidence level, indicating that differences between x-ray and NMR structures cannot account for the observed downfield 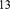
C chemical shift.

As predicted, a clear correlation is observed between structural regions consistent with an optimal 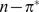 interaction and a downfield shift of the accepting carbonyl 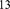
C resonance. Interestingly, the potential 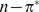 interactions were primarily observed between sequential (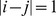) carbonyls. Out of the 164 structures and 2,516,360 residue pairs, only four pairs of carbonyls exhibited a through-space (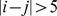) arrangement consistent with an optimal 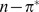 interaction. This result implies any protein stabilization energy obtained from the proposed 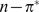 interaction is opportunistic, as opposed to a driving force for protein folding. Apparently, the formation of through-space 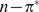 interactions is simply less favorable than for other interactions, such as hydrogen bonds or salt-bridges. This also implies that the predicted energy of 5.4 kJ/mol for an optimal 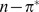 interaction is an over-estimate.

In actuality, an 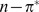 interaction that imparts a stability of 5.4 kJ/mol would likely fix adjacent pairs of carbonyl groups to preferred torsional angles in order to maximize this interaction. Correspondingly, the existence of these highly energetic 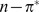 interactions would likely be detrimental to properly folding a protein structure. Folding a protein to its native fold would require distorting the majority of carbonyl pairs away from the ideal torsion angles for a proper 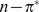 interaction. Only 12% (5,378 out of 45,792) of carbonyls from the 164 x-ray structures adopted conformations with optimal 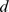 and 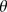 values for an 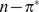 interaction. As a result, folding every protein structure would incur an initial energetic penalty of nearly 5.4 kJ/mol per carbonyl pair.

**Figure 2:**
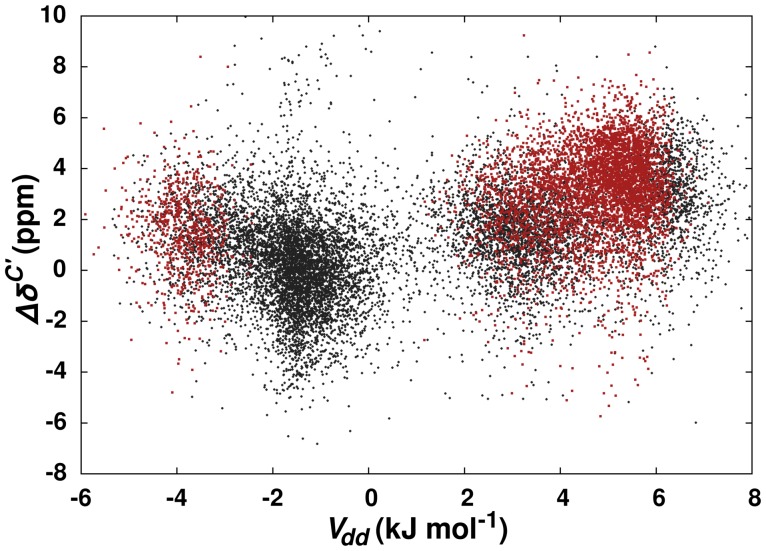
Carbonyl 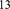
C Chemical Shifts and Dipole-Dipole Potential. Carbonyl 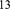
C chemical shift differences relative to random coil are plotted against calculated dipole-dipole potential (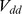). The dipole-dipole potential is calculated from the high-resolution x-ray structure using Equation 1. Pairs of carbonyls with 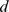 and 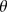 values within the optimal limits for an 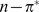 interaction are colored red.

A predominant number of the carbonyls consistent with an optimal 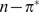 interaction and with a downfield shift of roughly 2.5 ppm fall within the typical 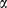-helical region of a Ramachandran plot, where the remaining residues are near the twisted 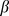-sheet region (see Figure S2). Significant chemical shift changes for carbonyl residues within secondary structures are well documented [19]. Previous analyses of structural factors contributing to carbonyl 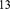
C chemical shifts have implicated hydrogen bond formation [20]–[22] or excluded hydrogen bond formation [23]–[25], have implicated 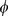, 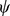, and 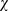 dihedral angles [24] or have excluded secondary structure parameters [20], [23]. Thus, other factors, such as hydrogen bonds or dipole-dipole interactions, may explain the apparent correlation between carbonyl 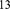
C shifts and the optimal 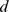 and 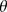 values for an 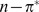 interaction. This is probable given the association of 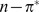 interactions with secondary structure elements. The contribution of a dipole-dipole interaction to carbonyl 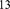
C chemical shifts is illustrated in Figure 2. The dipole-dipole potentials were calculated using the high-resolution x-ray structures for each of the 45,792 carbonyl pairs with a maximal distance of 6.0 Å between the donor oxygen and acceptor carbon. While there is significant scatter in the data, there is also a clear trend between a downfield carbonyl 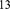
C chemical shift and an increasing dipole-dipole energy. Importantly, the cluster of acceptor carbonyls in Figure 2 with the largest 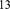
C chemical shift difference (3.15 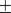 2.44 ppm) and positive dipole-dipole potentials also conforms to the optimal 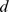 and 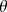 values for the predicted 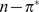 interaction.

**Figure 3:**
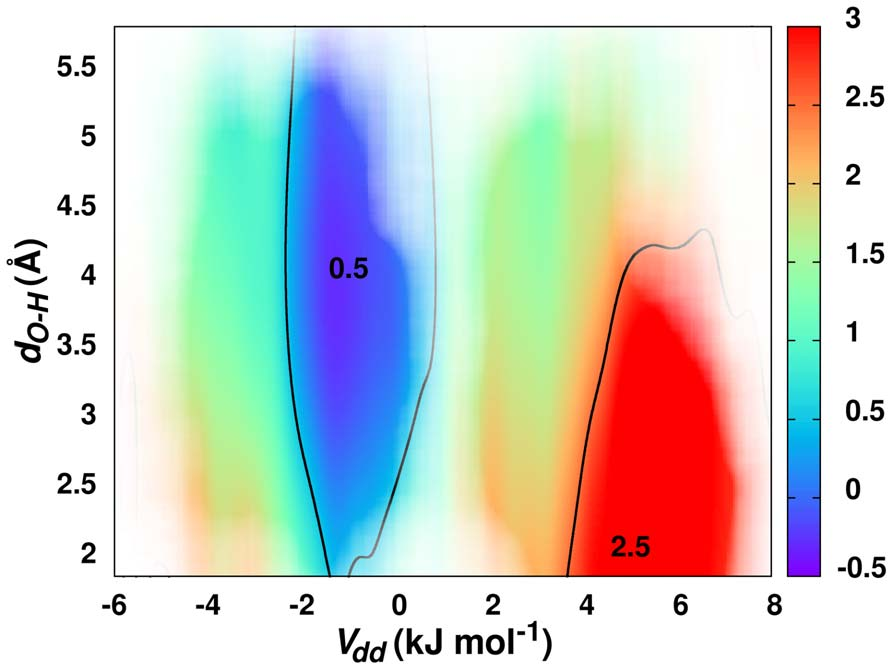
Carbonyl 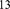
C Chemical Shifts and Hydrogen Bonds. Contour plot of 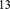
C carbonyl chemical shift differences as a function of calculated dipole-dipole potential (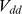) and calculated hydrogen bond length (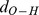). See also Figure S2.

The contribution of a hydrogen-bond interaction to the carbonyl 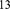
C chemical shift was similarly evaluated by calculating the shortest oxygen-hydrogen distance (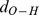) for each donor carbonyl. Again, the distances were calculated using the high-resolution x-ray structures for each of the 45,792 carbonyl pairs. A three-dimensional plot comparing the dipole-dipole potentials, oxygen-hydrogen distances, and the associated carbonyl 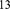
C chemical shifts is very revealing. It can be clearly seen from Figure 3 that any contribution from a hydrogen bond to the 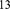
C carbonyl chemical shift is minimal relative to the dipole-dipole contribution. Both the 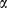-helical and 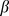-sheet regions, which obviously contain hydrogen bond interactions, have distinctly different 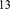
C carbonyl chemical shifts. The 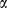-helical region corresponds to a positive dipole-dipole interaction, and correspondingly to a large carbonyl 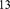
C chemical shift difference. Conversely, the 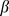-sheet region has a negative dipole-dipole interaction and a near zero carbonyl 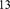
C chemical shift difference. These results further indicate a consistency with a dipole-dipole interaction as opposed to the predicted 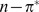 interaction.

It is important to note that there is a second cluster of carbonyls in Figure 2 with low 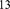
C chemical shifts and negative dipole-dipole potentials that are also consistent with the optimal 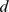 and 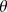 values for the predicted 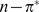 interaction. A visual inspection of the x-ray structures indicates that these carbonyl pairs are actually pointing away from each other and do not form the configuration for an 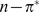 interaction illustrated in Figure 1A. Clearly, 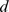 and 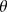 values alone fail to adequately define the optimal geometry of the dipole-dipole interaction that is apparently responsible for the observed downfield 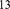
C chemical shifts.

**Figure 4:**
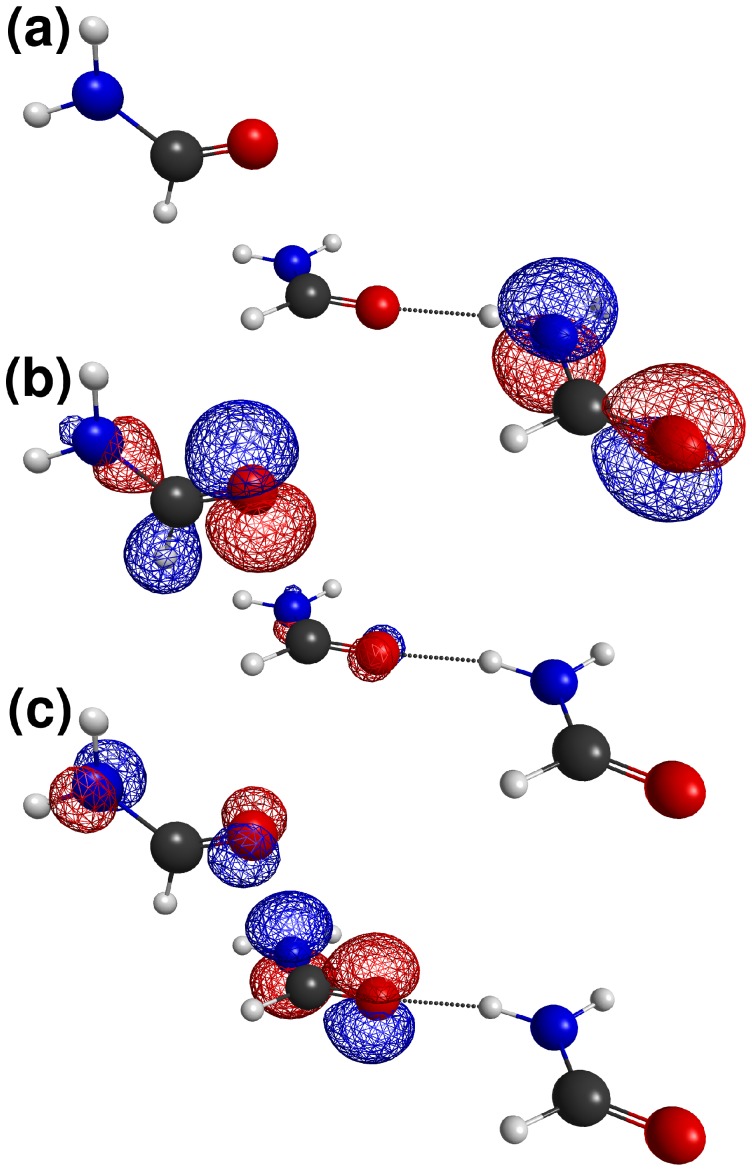
Formamide Trimer Model. Molecular orbitals of (**A**) the hydrogen bond donor, (**B**) the putative 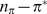 donor and (**C**) the putative 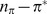 acceptor, in the trimeric complex used in quantum chemical calculations.

**Figure 5:**
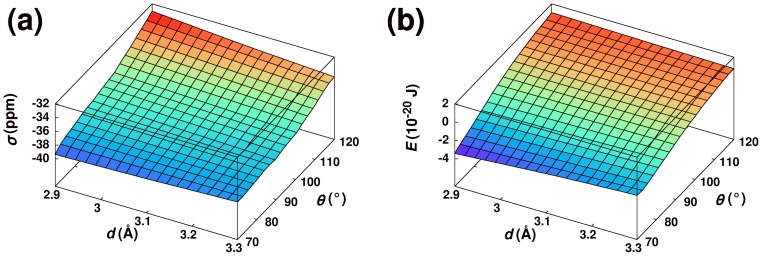
Summary of Quantum Chemical Calculations. Plot of calculated (**A**) carbonyl 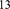
C chemical shielding (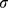) and (**B**) dipole-dipole interaction energy (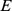) as a function of the distance between donor oxygen and acceptor carbon (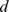) and the angle between carbonyl groups (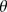). See also Figure S3.

To further examine the origin of these effects, quantum chemical calculations were conducted on a model system, a formamide trimer in which molecules 2 and 3 form an approximately planar, head-to-tail hydrogen bonded dimer, and molecule 3 acts as a putative 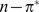 donor, with the 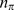 ‘donor’ oxygen fixed at a distance 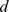 which ranges from 2.9 Å and 3.3 Å from the carbonyl carbon of molecule 2, with the O
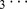
C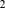 vector also fixed at angles 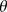 from 70° to 120° from the C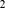 = O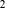 vector. To avoid problems with the use of density functional theory to model virtual orbitals, Möller-Plesset second order perturbation theory (MP2) was instead used, with a substantially larger basis set than in the previous work. The geometry and relevant Hartree-Fock orbitals of the complex used is shown in Figure 4, for 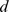 = 2.9 Å and 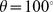. The computed chemical shielding is shown in Figure 5A as a function of 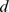 and 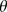. The shielding decreases monotonically with 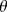, but, in contrast, the slope of the shielding surface with respect to 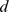 changes sign between 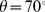 and 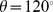. This shielding surface does not have the geometry expected if the chemical shielding dependencies on 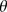 and 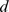 were dominated by an 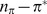 interaction, where shielding should be maximum at 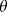 slightly larger than 90° and 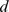 = 2.9 Å, decreasing rapidly with increasing values of 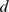.

However, the shielding surface does show a remarkable similarity to the dipole-dipole energy between the putative donor and acceptor, as shown in Figure 5B. This energy was computed using a very simple model assuming the electric dipole vector lies along the carbonyl bond for both molecules and has a value of 2.34 D or 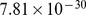 C

m. As can be seen, the dipole energy closely parallels the chemical shielding surface, monotonically increasing with 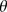 and inverting its slope with respect to 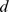 as 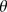 increases. This indicates the major influence on the carbonyl 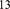
C chemical shielding is not an 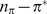 interaction but rather the electrostatic field from the neighboring carbonyl dipole. The correspondence is not, however, exact: the chemical shielding surface shows a small negative inflection around 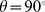, which is actually slightly reversed in the dipolar energy plot.

In order to examine whether an 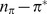 interaction might be responsible for this inflection, the chemical shielding surface was fit to a function proportional to the dipolar energy, under the assumption the dipole moment vector lies along the C = O bond direction, and the best fit subtracted from the chemical shielding surface (see Figure S3A). The residual shows a minimum at 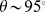, as would be expected for an 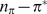 interaction, but the dip does not appear to decrease rapidly as 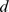 increases, as an orbital overlap term would. In fact, the residual is slightly larger at 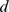 = 3.3 Å than at 2.9 Å (1.3 ppm vs. 1.1 ppm).

From the fit of the shielding surface to the estimated dipole interaction energy, with the assumption the magnitude of the electric dipole moment is that of a formamide monomer (3.7 D), a dependence of chemical shielding on field of 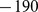 ppm/a.u. was obtained (1 atomic unit (a.u.) of electric field equals 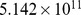 V/m). Direct calculations of the dependence of the shielding of an isolated formamide on an external applied field along the C = O bond direction gave a value of 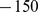 ppm/a.u. However, it is highly likely that this estimation of the dipole-dipole interaction for two amides is low. Firstly, higher electric multipole terms were neglected in the calculation, and these are likely to be substantial for a moiety as asymmetric as a peptide linkage, at these close proximities. Second, the interaction of the dipoles is likely to be enhanced by the highly polarizable hydrogen bond, which is necessarily omitted in the monomer model. Agreement of the model with direct estimates of the effect of electric field on shielding is therefore rather good.

The dependence of chemical shielding on hydrogen bonding strength for all combinations of 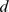 and 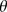 was examined as a function of the hydrogen bond distance 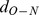 (see Figure S3B). In accordance with the results of Wishart and others [23]–[25], and contrary to initial naïve expectations, the effect was very small and independent of the position of the putative 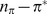 donor carbonyl.

**Figure 6:**
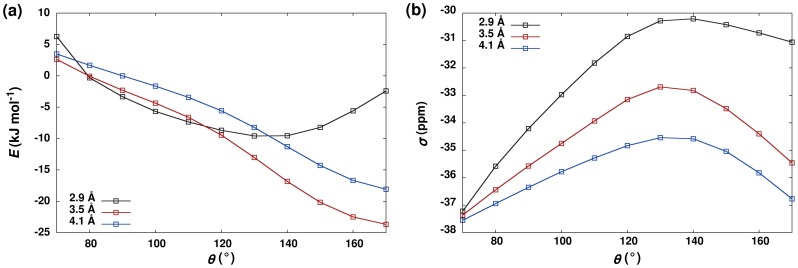
Summary of Quantum Chemical Calculations for ‘End-On’ Dipole Interaction. Plots of (**A**) interaction energy and (**B**) carbonyl 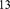
C chemical shielding (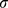) as a function of the angle between the carbonyls (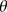) for three different distances (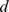) between the donor oxygen and acceptor carbon.

For the sake of completeness, the effect of an ‘end-on’ carbonyl-carbonyl interaction was examined, using a dimeric cluster in which the ‘donor’ carbonyl bond was parallel to the ‘donor’ oxygen-‘acceptor’ carbon vector, resulting in a possible 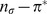 interaction. As can be seen in Figure 6, for values of 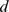 ranging from 2.9 Å to 4.1 Å, the chemical shielding also follows the negative of the dipolar interaction energy over the range 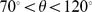, with little evidence of any effect of orbital overlap on chemical shielding.

One other outcome of the calculations is of possible note. While there was little discernible effect of the proposed 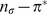 or 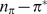 interactions on the shielding of the carbonyl carbon or the length of the carbonyl bond, substantial pyramidalization of the amide nitrogen was observed at low values of 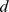 and values of 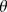 close to 90°. This would indicate that the primary effect of the ‘donor’ carbonyl might not be on the carbonyl 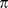 bond *per se*, but on its delocalization over the entire amide group. There was also a substantial lengthening of the carbon-nitrogen bond – consistent with a reduced bond order – accompanied by substantial changes in the computed 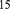
N chemical shielding. Thus, while no evidence was found of effects from 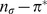 interactions on the 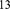
C NMR spectroscopy or the energetics of the system, such interactions might be detectable in 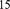
N chemical shifts. Unfortunately, 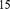
N shifts are known to be much more dispersed than carbonyl 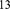
C shifts and are susceptible to a wide range of influences, so disentangling the interaction in real proteins might be a Herculean task.

## Discussion

When the molecular orbitals for the trimeric complex are examined in detail, the above results become clear. It is in fact misleading to think of amide groups as being dominated by the carbonyl 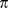 bond. The highest occupied molecular orbital (HOMO) of the formamide trimer in fact consists almost entirely of 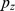 orbitals on the N and O, with wavefunctions of opposite sign. This is depicted in Figure 4A for the Hartree-Fock HOMO of the hydrogen bond donor (energy = 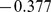 Ha). The orbital is slightly bonding with respect to the carbonyl, but the carbonyl carbon overall has very little contribution to the molecular orbital. The equivalent orbital of the putative acceptor (Figure 4B) has somewhat lower energy (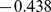 Ha) but shows remarkably little mixing with other molecular orbitals, and in particular little mixing with the 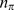 orbital of the putative 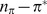 donor (Figure 4C). That orbital has in fact a very similar energy (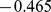 Ha), and at other geometries – specifically lower values of 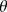, mixes with the HOMO of the acceptor. The reason for this is quite simple; because the HOMO has only a very small contribution for carbonyl carbon orbitals, bringing the 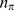 orbital closer to it has very little effect. The mixing that is present at smaller values of 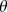 in fact seems to be partly responsible for the increased pyramidalization of the nitrogen of the acceptor at those orientations. We see no evidence of any orbital mixing that could be attributed to 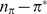 interactions. Given the weakness of the mixing with orbitals that are very close in energy to 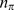 it is implausible that substantial mixing would be observed with an orbital almost a Hartree higher in energy.

In conclusion, quantum chemical calculations, experimental carbonyl 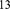
C chemical shifts and structural data indicate that a simple electrostatic dipole-dipole interaction explains the large downfield carbonyl 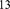
C chemical shift in an 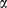-helix. There is no evidence for a significant contribution from an 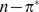 interaction to the carbonyl bond. The single indication of 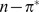 interactions seems to be a substantial lengthening of the carbon-nitrogen bond and pyramidalization of the nitrogen at 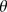 angles favorable for these interactions. In fact, such pyramidalization seems to be a logical consequence of the electronic structure of amides, whose 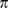 orbitals are delocalized over the whole system.

## Methods

### Analysis of Experimental Structures

A detailed statistical analysis was performed to correlate experimentally observed carbonyl 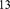
C chemical shifts with structural parameters between all possible pairs of carbonyls. Specifically, the angle between the carbonyls (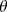) and the distance (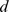) between the oxygen and carbon were compared to experimental carbonyl 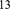
C chemical shifts. The PISCES [26] (http://dunbrack.fcc.edu/pisces) set of 2,885 high-resolution (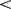
1.6 Å) x-ray crystal structures with less than 30% pairwise sequence identity selected from the RCSB Protein Data Bank (PDB) [27] was used for this analysis. Each structure was associated with assigned NMR 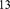
C and 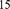
N chemical shifts from the Biological Magnetic Resonance Bank (BMRB: www.bmrb.wisc.edu) [28] by FASTA [29] sequence alignments. The best match with an E-value 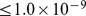 and sequence identity 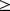
95% was chosen, where the median E-value was 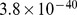. The aligned 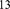
C and 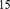
N chemical shifts were uniformly referenced with the SHIFTCOR software tool [30]. The protein interfaces, surfaces and assemblies software tool (PISA, http://www.ebi.ac.uk/pdbe/prot_int/pistart.html) [31] was used to predict residues involved in crystal packing interfaces. Residues with B-factors two standard deviations from the mean within each structure were identified as dynamic. Also, 3,699 NMR solution structures with PDB depositions cross-linked to the BMRB were used as a validation dataset, with alignments performed in an identical fashion to the analyzed x-ray structures.

A set of Perl scripts was written to extract structural parameters from the x-ray structures. For each structure in the selected set, all pairs of residues were analyzed for the possibility of an 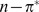 interaction. Values of 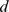 and 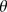 were calculated for each residue pair, and torsional angles 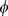 and 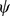 were calculated for the ‘acceptor’ residue of each pair. Pairs of carbonyls with 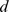 and 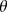 values within the optimal limits for an 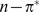 interaction were labeled as interactors (see Figure S1). Standard random-coil chemical shifts were subtracted from the experimental carbonyl 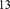
C chemical shifts for each residue.

For all pairs of residues, a dipole-dipole potential (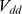) was calculated from the high-resolution x-ray structures using equation 1:


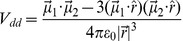
(1)

where 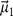 and 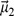 are the two C = O bond vectors, 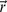 is the vector between the centers of the C = O bonds, and 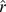 is its unit vector. The nominal value of 2.34 Debye was taken for the carbonyl dipole moment. Similarly, for all residue pairs, the minimum possible hydrogen bond length (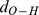) was calculated from the high-resolution x-ray structures. Hydrogen bond lengths were calculated based on the nearest non-neighboring backbone amide hydrogen, with a maximal bonding angle of 60°.

### Model Compound Calculations

Quantum chemical calculations were done using the program Gaussian-09 [32]. A nearly planar formamide head-to-tail dimer, composed of a formamide monomer (molecule 1) hydrogen bonded through its C = O group to the N-H group of a second, nearly parallel formamide (molecule 2) was chosen to approximate the hydrogen bonding motif found in both 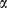-helices and antiparallel 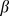-sheets. The dimer was fully optimized at the MP2/6-311++G(2d,p) level; Möller-Plesset second order perturbation theory (MP2) was chosen because it is superior in modeling long-range and dispersive contributions to the electron correlation Hamiltonian. A third formamide (molecule 3) was then added to generate the putative 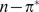 interaction with molecule 1, imposing the following constraints: (1) C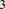 = O
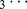
C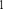 angle fixed at 90°, to ensure the 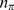 orbital of molecule 3 points toward the carbonyl of molecule 1 (2) O
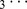
C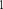 = O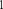 constrained to a set of fixed angles 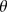, ranging from 70° to 120° (3) 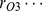
C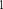 constrained to a set of fixed distances 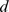, ranging from 2.9 Å to 3.3 Å (5) 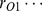
N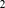 constrained to a set of fixed distances, ranging from 2.8 Å to 3.2 Å, to vary the strength of the hydrogen bond. The system of three molecules was then subjected to constrained optimization at the same level as before. The optimized trimolecular system at an angle 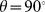 is shown in Figure 5. Finally, a full set of shielding tensors was computed using standard GIAO methods.

## Supporting Information

Figure S1

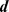
 **,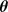-space Analysis of Experimental Structures.** Plot of the distance (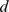) and angle (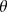) measured between each of the 45,792 pairs of carbonyls with a potential 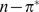 interaction. The relative density of points in the occupied 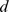 and 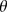 space was used to generate a transparency mask for Figure 1.(TIF)

Figure S2
**Ramachandran-space Analysis of Experimental Structures.** Ramachandran plot of carbonyls with 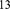
C chemical shift differences relative to random coil that are 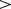
2.5 ppm. The acceptor carbonyls from each pair of carbonyls with 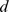 and 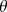 values within the optimal limits for an 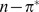 interaction are colored red.(TIF)

Figure S3
**Supplemental Quantum Chemical Calculation Plots.** (**A**) Plot of the residuals for the fit of the chemical shielding surface to a function proportional to the dipole-dipole energy. (**B**) Summary of the quantum chemical calculations of the hydrogen bond contribution to the dipole-dipole interaction; plot of carbonyl 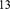
C chemical shielding (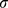) as a function of the hydrogen bond angle (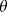) and distance (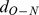).(TIF)
