# Supplementary figures and images for: 13C NMR Reveals No Evidence of n−π* Interactions in Proteins
Source: PLoS One. 2012 Aug 2;7(8):e42075. doi: 10.1371/journal.pone.0042075 (PMC3410932; doi:10.1371/journal.pone.0042075)

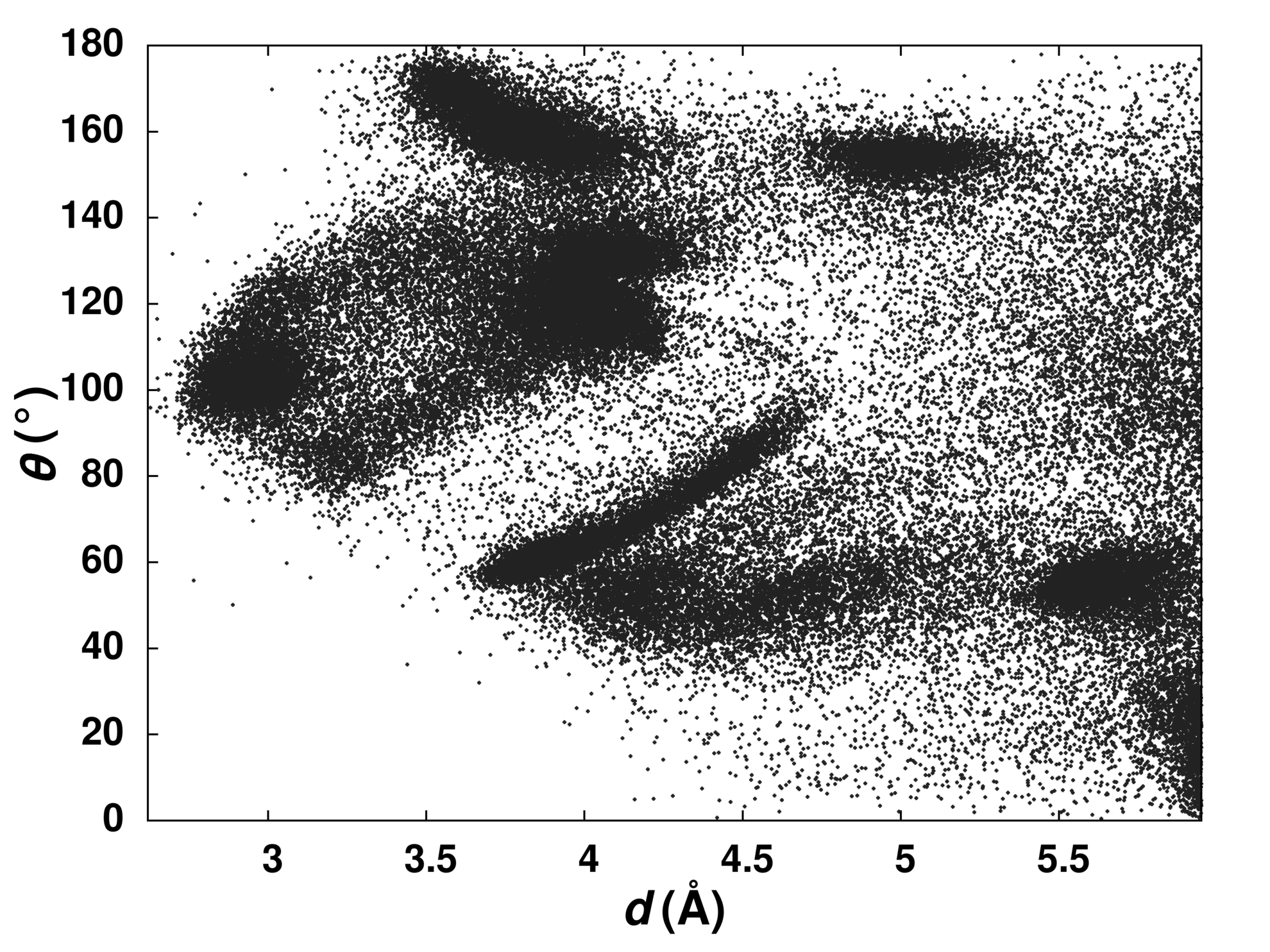

Supplement: Figure S1 — ,-space Analysis of Experimental Structures. Plot of the distance () and angle () measured between each of the 45,792 pairs of carbonyls with a potential interaction. The relative density of points in the occupied and space was used to generate a transparency mask for Figure 1. (TIF) [file pone.0042075.s001.tif]

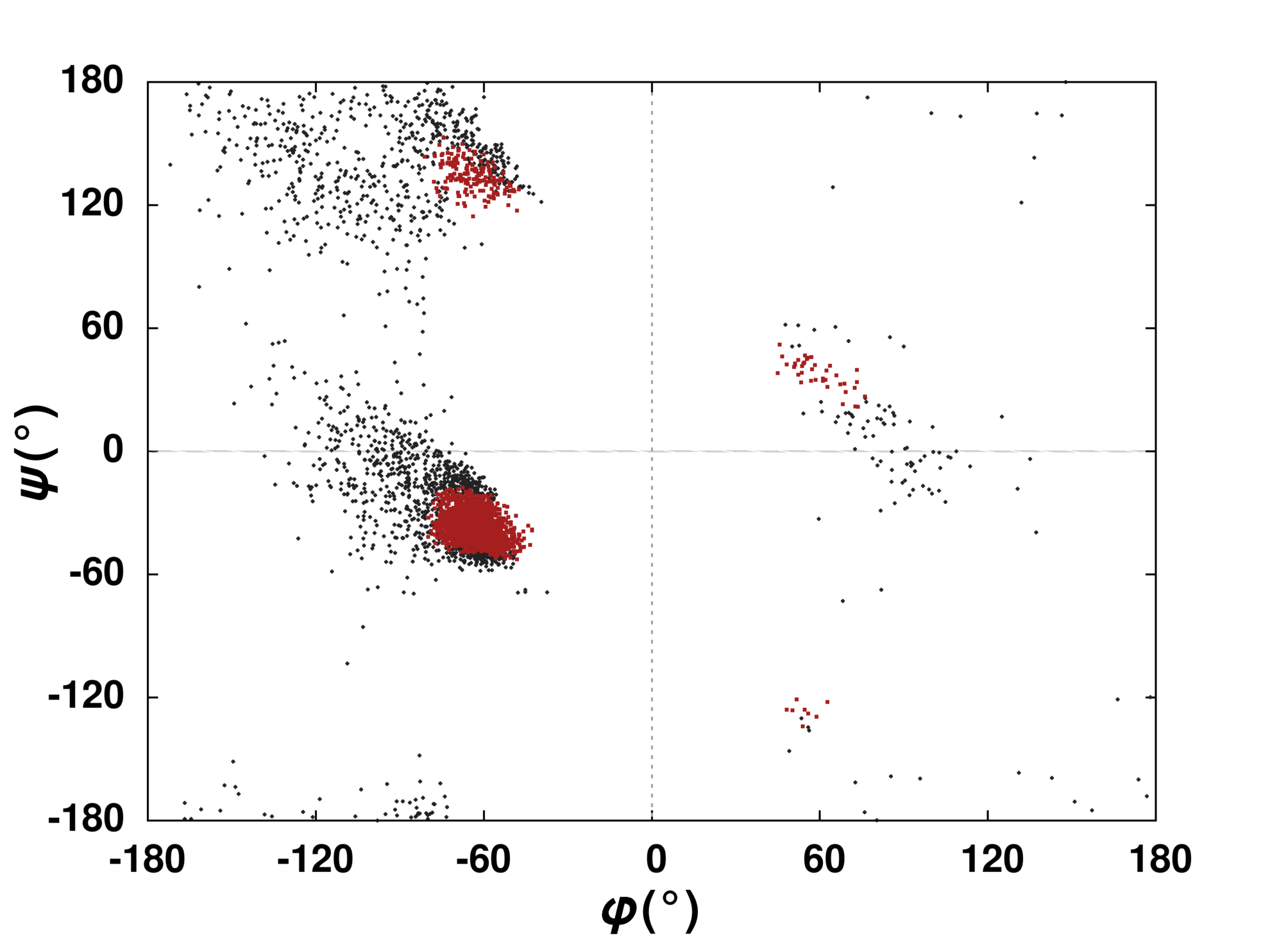

Supplement: Figure S2 — Ramachandran-space Analysis of Experimental Structures. Ramachandran plot of carbonyls with C chemical shift differences relative to random coil that are 2.5 ppm. The acceptor carbonyls from each pair of carbonyls with and values within the optimal limits for an interaction are colored red. (TIF) [file pone.0042075.s002.tif]

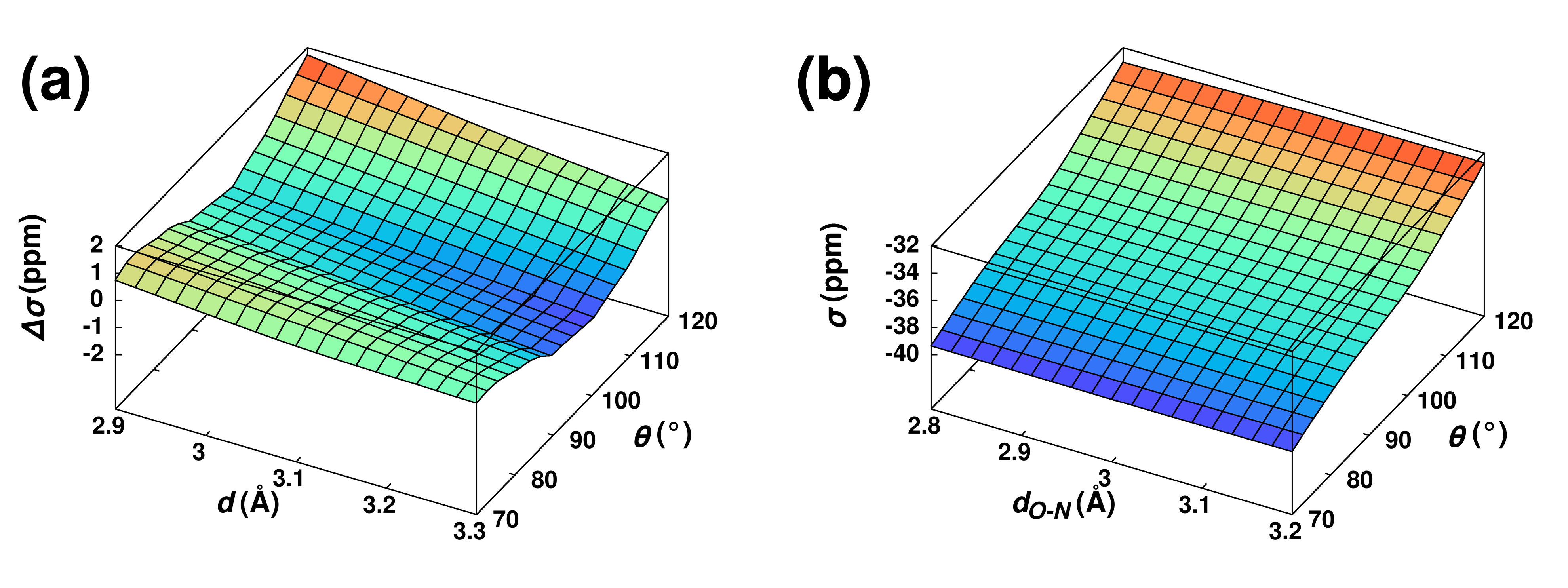

Supplement: Figure S3 — Supplemental Quantum Chemical Calculation Plots. (A) Plot of the residuals for the fit of the chemical shielding surface to a function proportional to the dipole-dipole energy. (B) Summary of the quantum chemical calculations of the hydrogen bond contribution to the dipole-dipole interaction; plot of carbonyl C chemical shielding () as a function of the hydrogen bond angle () and distance (). (TIF) [file pone.0042075.s003.tif]
